# Can Wearable Technology Help Guide Dieting Safety?

**DOI:** 10.21203/rs.3.rs-5619684/v1

**Published:** 2024-12-12

**Authors:** Maya Tang, Joseph Powell, Xiao Li

**Affiliations:** Hathaway Brown School; Case Western Reserve University; Case Western Reserve University

**Keywords:** dieting, wearable technology, resting heart rate

## Abstract

**Background:**

The safety of dietary interventions is often unmonitored. Wearable technology can track elevations in resting heart rate (RHR), a marker of physiologic stress, which may provide safety information that is incremental to self-reported data.

**Methods:**

A single subject was placed on an isocaloric diet for four weeks. In weeks # 1 and 4, timing of food consumption was unregulated. In week #2, food was consumed during a three-hour feeding window (one-meal-a-day, OMAD). During week #3, food was consumed at six intervals, spaced three hours apart (6-meal diet). A Fitbit Versa^™^ was worn continuously, and questionnaires were administered twice daily.

**Results:**

Meal frequency did not affect the subject’s weight. Hunger scores from morning and night were widely split on OMAD and relatively constant on the 6-meal diet. Energy, happiness, irritability, and sleep scores were more favorable on the 6-meal diet than on OMAD. RHR extracted from the wearable device was lower during the 6-meal diet than during OMAD, especially in the late afternoon, evening, and nighttime (p<0.05). Lower RHR during the 6-meal diet corresponded to more favorable questionnaire scores.

**Conclusions:**

Changes in RHR patterns acquired by wearable technology are promising indicators of physiologic stress during dietary interventions. Wearable technology can provide physiologic data that are complementary to questionnaire scores or timed manual measurements.

## 1. Introduction

Dieting is primarily initiated as a means to promote weight loss. However, more recently, multiple diets have been promoted as providing additional health benefits, independent of weight loss [1]. For example, the intermittent fasting diet is a popular contemporary diet that has been studied in both animals and humans. This diet has been shown to have a metabolic and mortality benefit in animal models [2, 3]. It has beneficial effects on human metabolism, including a positive impact on obesity, hypertension, insulin resistance, and inflammation [4–6]. In addition, it has been shown to lower cardiovascular risk and cancer risk [1, 7]. The grazing diet, which consists of several small meals a day, has not been studied as well in the scientific community [8]. However, it is a very popular diet that receives a lot of attention on the internet.

The impact of these types of diets on any given individual’s physiology is not well ascertained. Wearable technology provides an interesting method to track physiologic parameters such as heart rate, heart rate variability, activity tracking, and sleep tracking. Wearable devices have been used to monitor resting heart rate, which has been validated as a marker of both physiologic and mental stress [9, 10, 11, 12]. The impact of dietary changes on physiologic markers of stress has not been well studied. Wearable technology provides a potential tool to track the impact of dieting on physiologic markers of stress in real time while an individual is making a dietary change [13]. If wearable technology proves to be useful in monitoring individuals during dietary changes, this information can be incorporated into dieting applications that help individuals track the success and safety of their diet plans. The aim of this study is to examine the effect of meal frequency on well-being and stress, using a combination of questionnaires, manually obtained vital signs, and physiologic data from wearable devices. If alteration of meal frequency in an isocaloric diet induces physiologic stress, then resting heart rate will increase. Furthermore, wearable technology data will provide incremental benefit in monitoring the safety of diets above questionnaire-based data.

## 2. Materials and Methods

### Patient Information

This pilot study was conducted on a single study participant and was approved by the Case Western Reserve University’s Institutional Review Board, STUDY20240653. The study subject was a 48-year-old female with no underlying health conditions. The study subject signed voluntary informed consent to participate in a dietary intervention study utilizing wearable technology and manually collected data from questionnaires and vital sign measurements. For one month prior to collection of data, the study subject ceased consumption of all caffeinated foods and beverages. Furthermore, prior to data collection, the study subject’s usual diet was recorded, and the average daily caloric intake was used to construct the study diet.

### Study Diet

The study diet was isocaloric and consisted of the following items: 1 string cheese, 1 low-fat yogurt, 75 grams of a broccoli, cauliflower, carrot vegetable blend, 1 jam sandwich (2 slices Italian bread, 1 tbsp strawberry jam, ½ tbsp butter), 1 Stouffer’s^®^ Cheese French Bread pizza, and 5 cups of herbal non-caffeinated tea (no sugar or additives allowed).

### Diet Administration

Week #1 of the study was a run-in control period to ensure reliable and consistent acquisition of data. During week #2, the study subject consumed the study diet in one-meal-a-day (OMAD), timed between 4–7pm. In week #3, the study diet was consumed over 6 time-intervals, spaced 3 hours apart (7am, 10am, 1pm, 4 pm, 7 pm, and 10pm). Week #4 was the second, wash-out, control period. During weeks #1 and 4, the timing of the study participant’s meals was not pre-specified, but the isocaloric diet was maintained. The timing of study beverages was not regulated during the study in order to avoid dehydration.

### Manual Data Collection

The study participant collected data manually at 7am and 7pm. This data included vital sign information: systolic blood pressure (mm Hg), diastolic blood pressure (mm Hg), heart rate (bpm), oxygen saturation (%), and weight (lbs). Blood pressure and heart rate information were obtained using an automated blood pressure cuff placed on the upper arm. Oxygen saturation was obtained from a pulse oximeter placed on the subject’s finger. Weight was recorded from a digital scale. Well-being was gauged by a series of self-reported scales (Table 1). Sleepiness was assessed by two established sleep scales: the Epworth Sleepiness Scale (ESS) [14]

### Wearable Data Collection

A wearable device (Fitbit Versa^™^) was worn continuously during the study period, with the exception of a brief daily charging period that coincided with the study subject’s morning shower. Resting heart rate data was extracted from the wearable devices. Wearable data from minute time points was plotted over 24 hours and plotted on an hourly basis with box and whisker plots with a 1.5 interquartile range as the whisker boundary. Data were also analyzed in specific time periods: 7am to 7pm, 7pm to 7 am, 3pm to 11pm, and 11pm to 7am.

### Statistical Analysis

Wilcoxon Rank Sum tests were used to compare the significance of the difference in resting heart rate between OMAD and the 6-meal diet, with p < 0.05 as the determinant of significance. All analyses were performed using R version 4.2.2 and Matlab R2019a.

## 3. Results

Manually recorded vital signs, averaged for each week of the study period, are shown in Table 2. Heat rate data showed variation between morning and evening. Weight also fluctuated during the day but did not change substantially during the study period.

**Figure 1:**
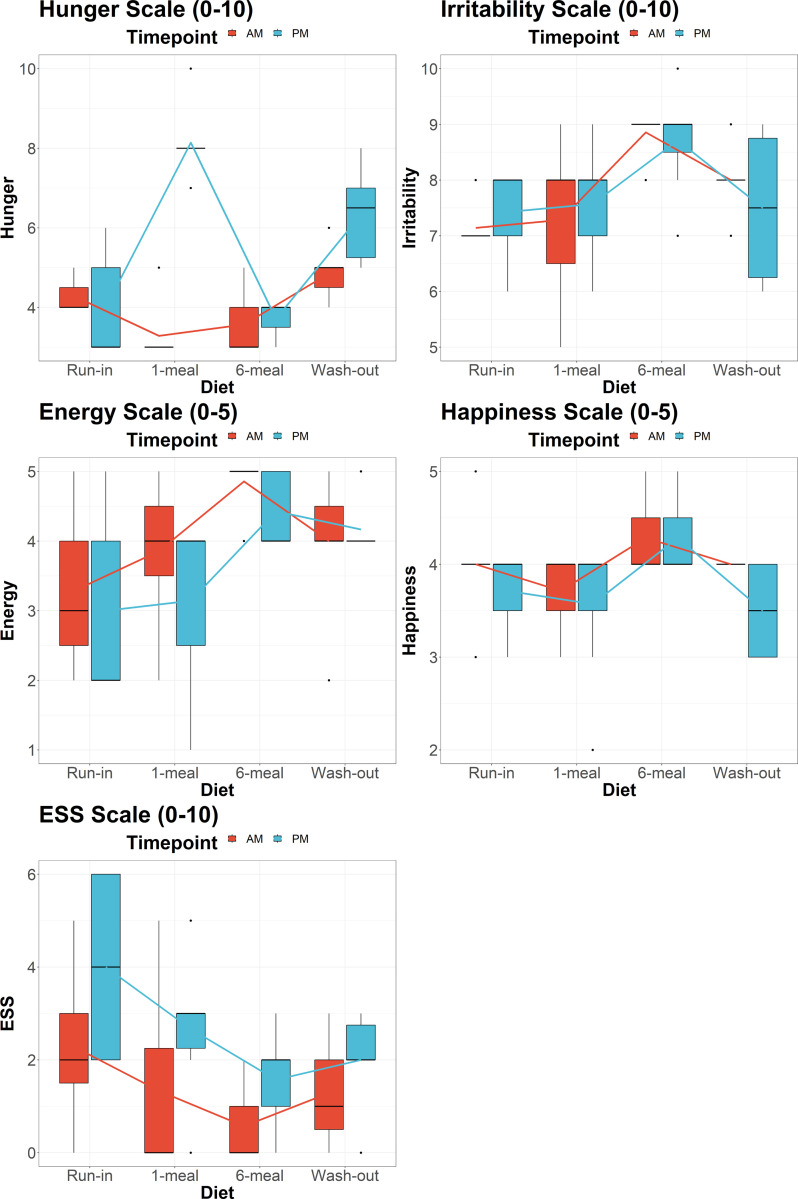
Quality of life data from self-reported questionnaires over study periods.

Quality of life data from self-reported questionnaires are shown in Figure 1. Hunger scores were widely split on OMAD (hungry in the morning and very full in the evening). In contrast, during the 6-meal diet, hunger scores were relatively constant. Energy, happiness, and irritability scores were more favorable on the 6-meal diet than on OMAD. Sleepiness was greater in the evening during OMAD than the 6-meal diet.

**Figure 2:**
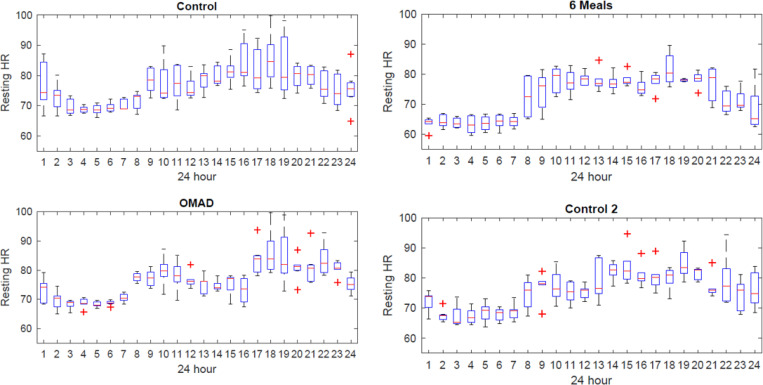
Resting heart rate from wearable device over a 24-hour period.

Resting heart rate over a 24-hour period, extracted from the wearable device, is shown in Figure 2.

**Figure 3:**
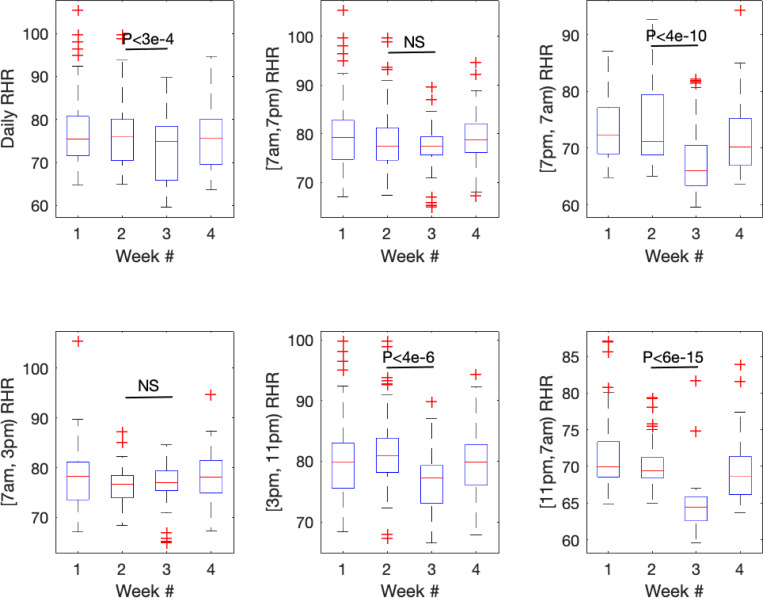
Wearable analysis of resting heart rate at specific time intervals of study periods.

Resting heart rate appeared overall lower during evening/night hours during the 6-meal diet. There were more oscillations in heart rate during the OMAD diet. The difference in resting heart rate between OMAD and the 6-meal diet was analyzed in 6 time periods: 24-hour (daily), 7am-7pm, 7pm-7am, 7am-3pm, 3pm-11pm, 11pm-7am (Figure 3).

## 4. Discussion

Two contemporary, popular diets were assessed for their effects on subjective well-being and objective changes in resting heart rate (RHR). In the literature, the intermittent-fasting diet has been touted to have many health benefits beyond its effect on weight loss. One-meal-a-day (OMAD) is an extreme form of the intermittent fasting diet, during which all food is consumed in a very restricted time interval once a day. Other forms of intermittent fasting include a broader feeding interval of 8–10 hours a day or fasting for 2/7 days per week. In contrast, the grazing diet consists of several small meals a day. The impact of meal frequency on subjective and objective measures of well-being is not well reported.

This study demonstrates that subjective scores of well-being were higher during the 6-meal diet intervention than during OMAD. More frequent meal timing led to greater energy and happiness, and less irritability and sleepiness. Interestingly, these subjective perceptions of well-being corresponded to an objectively lower RHR collected by wearable devices. Elevations in RHR have been shown to be indicative of physiologic and psychologic stress [9,10]. As such, this n=1 study indicated that for this study participant, the 6-meal diet produced less stress and greater well-being than the OMAD diet. While previous studies may be able to demonstrate the health benefits of a particular diet in a large population, wearable devices may be able to provide direct measures of the impact of a particular diet on an individual. This type of information may be useful in tailoring a diet plan to individuals with different metabolic needs and different underlying health conditions.

Wearable devices are capable of collecting heart rate data continuously. This feature was instrumental in detecting a difference in the RHR between the 2 diets. In fact, the greatest impact on RHR was seen during sleeping hours. The manually collected heart rates at 7am and 7pm were an insensitive measure of the impact of diet on resting heart rate. This may be explained by the observation from the wearable devices that heart rate fluctuated significantly during different time periods of the day. Heart rate data collected manually at 2 time points may be influenced by the time of day, activity, position, proximity to meal consumption, and random sampling errors. Moreover, heart rate during sleep is completely missed by manual collection. This time interval, which had the least variability in heart rate, may be a very useful indicator of the impact on diet on physiologic stress. The wearable devices also showed some oscillations in heart rate during a 24-hour period, especially during the OMAD diet. The impact of diet on circadian rhythms is another physiologic parameter that can be captured by wearable devices with continuous heart rate monitoring.

At this time, many diet plans utilize apps on smart phones in order to help people monitor their progress on the diet [11]. People can record information such as food intake, weight, and exercise times. Calorie and nutrition information is built-in to many of these apps, which helps to maintain a consistent and balanced diet. Wearable devices show promise in being able to integrate with diet apps to provide physiologic data that could guide the safety of the diet.

This study was a pilot study that had several limitations. The study was conducted on a single individual over a 1-month study period. Since the study diet was highly regulated and was tailored to the study subject’s usual daily caloric intake, the pilot study was most feasible with a single subject. Single subject studies can be very useful in precision medicine. They help us to generate hypotheses and can demonstrate the effects of an intervention on an individual. Larger studies will likely require some tailoring of study diets to individual preferences and tolerability. This was particularly evident in the OMAD diet. Consumption of an entire day’s calories in a 3-hour time window had physical limitations that may vary from one individual to another. Each diet was maintained for a period of 1 week. In future studies, the diet duration would ideally be longer (e.g. 1–2 months) with a longer wash out period in between dietary interventions. It was not easy for the study participant to maintain the diet employed in this study for long periods of time due to the highly restricted food choices. If a study of longer duration is conducted, it may be necessary to offer study participants a greater variety of foods that still achieve an isocaloric diet. The reproducibility of the data observed in this study will need to be measured. The reproducibility of the findings using 2 distinct wearable devices and repeated tests of the same device would also increase the accuracy of the data.

## 5. Conclusion

Changes in patterns of resting heart rate acquired by wearable technology are promising indicators of physiologic stress during dietary interventions. Data from this pilot study support the hypothesis that physiologic stress induced by alterations in meal-timing could be detected by an increase in resting heart rate. Furthermore, wearable devices provide physiologic data about the safety of dietary interventions that are complementary to information obtained by questionnaires and times manual measurements. Although further studies are needed, hopefully, in the future, data from wearable devices can be coupled with information in dieting/fitness apps to help individuals monitor the safety of their diet plans.

## Figures and Tables

**Table 1:** Scales for self-reported questionnaires.

Score	Hunger	Irritability	Energy	Happiness
1	dizzy, nauseated, ill	Stays angry contstantly	No energy	Very unhappy
2	extremely hungry	Stays angry for long periods of time	Tired, able to do few tasks	Mildly unhappy
3	Hungry, stomach growling	Angry for short periods of time	Not tired, lacking motivation	Neutral
4	“I could eat”	Loses temper easily	Mildly energetic	Mildly happy
5	Not full but not hungry - neutral	Loses temper when mildly provoked	Very energetic	Very happy
6	Full stomach but not satisfied	Loses temper when heavily provoked		
7	Satisfied	Calm and positive most of the day		
8	Uncomfortably full	Calm and positive throughout the day		
9	Stuffed, very uncomfortable	Remains calm and positive when mildly provoked		
10	Physically ill, nauseous, sick	Remains calm and positive even when heavily provoked		

**Table 2.** Averaged vital signs during study periods.

	Systolic BP (mmHg)	Diastolic BP (mmHg)	Heart rate (bpm)	Oxygen Saturation (%)	Weight (lb)
Control 7am	94	61	89	98	93.9
Control 7pm	102	62	78	99	95.7
OMAD 7am	98	64	97	98	92.1
OMAD 7pm	105	65	83	99	94.3
6 meal 7am	96	63	101	98	91.5
6 meal 7pm	104	64	76	99	94.2
Control-2 7am	94	62	99	98	92.2
Control-2 7pm	106	66	70	100	94.9

1 Abbreviations: BP, blood pressure; OMAD, one meal a day.

## Data Availability

The data that support the findings of this study are available from the corresponding author upon reasonable request.
